# Medical and Philosophical News

**Published:** 1781-03

**Authors:** 


M. Gerbier, a French phyfician, having invented a remedy for cancers, it has been lately adminiftered to feven patients at Paris, under the direction of M. Romilais, one of the phyficians to to the Hotel Dieu. The refults of thefe trials were, 1. That only one patient feemed to be cured. 2. That two were fomewhat relieved, though far from being cured. 3. That a fourth patient who was afflicted with fcurvy, as well as cancer, was much worfe after taking it; his fcorbutic fymptoms, though very flight at firft, having made a rapid progrefs after he took the medicine, without the cancers being at all relieved by it. 4. That two other patients died, one foon, and the other five months after taking the remedy, though without its appearing that the medicine was in any degree the caufe of their death. 5. That one of the patients feemed to have fallen a victim to her courage, in perfevering in the ufe of a remedy, which from the firft produced only the moft melancholy effects.

This medicine, we are told, proves violently emetic, an effect that might naturally be expected from a remedy compofed chiefly of verdigreafe.

